# Altering 15‐Lipoxygenases to 18‐Lipoxygenases and Their Application to the Production of 5,18‐Dihydroxyeicosapentaenoic Acids

**DOI:** 10.1002/bit.28995

**Published:** 2025-04-16

**Authors:** Jin Lee, Su‐Hwan Kang, Tae‐Eui Lee, Deok‐Kun Oh

**Affiliations:** ^1^ Department of Bioscience and Biotechnology Konkuk University Seoul South Korea

**Keywords:** 18‐dihydroxyeicosapentaenoic acids, 5,18‐hydroxyeicosapentaenoic acids, biotransformation, engineered 18‐lipoxygenases, resolvin E2, structure‐guided enzyme engineering

## Abstract

Resolvin E2 (RvE2), 5*S*,18*R*‐dihydroxyeicosapentaenoic acid (5*S*,18*R‐*DiHEPE), and 18*S*‐RvE2 (5*S*,18*S*‐DiHEPE) are specialized pro‐resolving mediators that function in the resolution of inflammation. These SPMs have been produced in trace amounts from eicosapentaenoic acid (EPA) using acetylated cyclooxygenase‐2 or cytochrome P450 and 5‐lipoxygenase (5‐LOX) via 18*R*‐ and 18*S*‐hydroxyeicosapentaenoic acid (18*R*‐ and 18*S*‐HEPE) intermediates. In this study, we engineered 15*R*‐LOX from *Sorangium cellulosum* and 15*S*‐LOX from *Archangium violaceum* into 18*R*‐LOX (L423W/L424M/L568M variant of 15*R*‐LOX) and 18*S*‐LOX (L429W/L430M/L575M variant of 15*S*‐LOX), respectively, via structure‐guided enzyme engineering. The engineered 18*R*‐LOX converted EPA into 72.5% 18*R*‐HEPE and 27.5% 15*R*‐HEPE, while the engineered 18*S*‐LOX formed 81.8% 18*S*‐HEPE and 18.2% 15*S*‐HEPE. *Escherichia coli* expressing the engineered 18*R*‐ or 18*S*‐LOX converted 4.0 or 3.0 mM EPA into 2.0 mM (641 mg/L) 18*R*‐HEPE or 1.8 mM (577 mg/L) 18*S*‐HEPE in 20 min, respectively, achieving concentrations that were > 10^5^‐fold higher than those reported previously. Furthermore, 5*S*‐LOX from *Danio rerio* (zebrafish) converted a concentration of 0.5 mM of the prepared 18*R*‐ or 18*S*‐HEPE into 0.24 mM (81 mg/L) RvE2 or 0.22 mM (74 mg/L) 18*S*‐RvE2 in 30 min, respectively. To the best of our knowledge, this represents the first identification of 18‐LOXs and first qualitative production of RvE2 and 18*S*‐RvE2.

## Graphical abstract

15*R*‐ and 15*S*‐lipoxygenases (15*R*‐ and 15*S*‐LOXs) were engineered into 18*R*‐ and 18*S*‐LOXs, respectively. *Escherichia coli* expressing these enzymes converted eicosapentaenoic acid into 18*R*‐ and 18*S*‐hydroxyeicosapentaenoic acids (18*R*‐ and 18*S*‐HEPEs), respectively. 5*S*‐LOX converted 18*R*‐ and 18*S*‐HEPEs into resolvin E2 (RvE2), 5*S*,18*R*‐dihydroxyeicosapentaenoic acid (5*S*,18*R*‐DiHEPE), and 18*S*‐RvE2 (5*S*,18*S*‐DiHEPE), respectively.

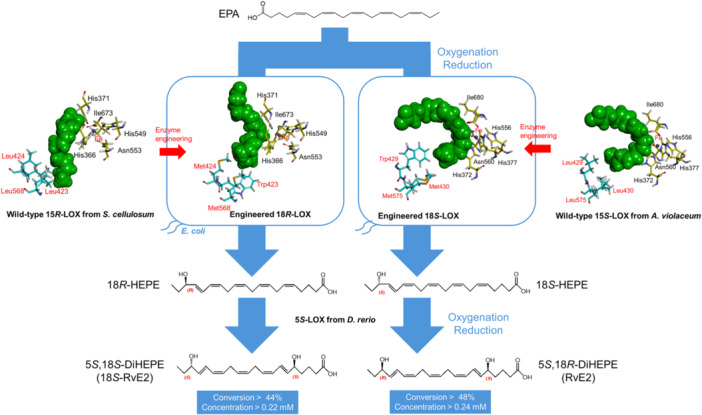

## 1 Introduction

Specialized pro‐resolving mediators (SPMs) are generated in trace amounts from endogenous polyunsaturated fatty acids (PUFAs) by polymorphonuclear leukocytes and M2 macrophages in the inflammatory and infection environment of humans (Serhan 2014). These mediators play a pivotal role in the removal of bacteria, viruses, dead cells, and debris as well as in the maintenance of homeostasis, the remission of fever, the maintenance of vascular integrity and perfusion, the regeneration of damaged tissue, and the alleviation of pain; thus, promoting the resolution of inflammation and infection (Basil and Levy 2016). Consequently, SPMs have received great attention as potential therapeutic agents.

SPMs are classified into the lipoxin (LX), protectin, marmesin (MaR), and resolvin (Rv) families (An et al. 2021). The Rv family is further subdivided into resolvin E (RvE), D, and T series, derived from eicosapentaenoic acid (EPA), docosahexaenoic acid (DHA), and n‐3 docosapentaenoic acid (n‐3 DPA), respectively. The RvE series includes 5*S*,12*R*,18*R*‐trihydroxyeicosapentaenoic acid (5*S*,12*R*,18*R*‐TriHEPE, RvE1), 5*S*,12*R*,18*S*‐TriHEPE (18*S*‐RvE1), 5*S*,18*R*‐dihydroxyeicosapentaenoic acid (5*S*,18*R*‐DiHEPE, RvE2), 5*S*,18*S*‐DiHEPE (18*S*‐RvE2), 17*R*,18*R*‐DiHEPE (RvE3), 17*R*,18*S*‐DiHEPE (18*S*‐RvE3), 5*S*,15*S*‐DiHEPE (RvE4), and 5*R*,15*R*‐DiHEPE (5*R*,15*R*‐RvE4) (Serhan et al. 2022). RvE4 and 5*R*,15*R*‐RvE4 have been quantitatively produced from EPA using double‐oxygenating 15*S*‐ and 15*R*‐lipoxygenases (LOXs), respectively (J. Lee et al. 2020; J. Lee et al. 2024a). The generation of RvE2 and 18*S*‐RvE2 through aspirin‐treated (acetylated) cyclooxygenase‐2 (COX‐2) or cytochrome P450 (CYP450) with 5*S*‐LOX from EPA has been confirmed (Oh et al. 2011). However, their quantitative production has not been achieved because of the low efficiency of these enzymes.

In contrast to COXs and CYP450s, LOXs exhibit distinct stereoselectivity and positional selectivity because of their specific substrate‐binding and site‐selective peroxidation. LOXs are promising enzymes, capable of producing hydroxy fatty acids at high concentrations from C20‐ and C22‐PUFAs under reduction conditions. Previous studies have demonstrated the qualitative conversion of EPA into positional‐selective 5*S*‐, 8*S*‐, 8*R*‐, 9*S*‐, 11*S*‐, 11*R*‐, 15*S*‐, and 15*R*‐hydroxyeicosapentaenoic acids (HEPEs) through 5*S*‐ (Shin et al. 2023), 8*S*‐ (Shin et al. 2019), 8*R*‐ (T. E. Lee et al. 2024b), 9*S*‐ (M. J. Kim et al. 2023; S. E. Kim et al. 2022b), 11*S*‐ (An and Oh 2018b), 11*R*‐ (Gao et al. 2010; S. E. Kim et al. 2022a), 12*S*‐ (An et al. 2018a; T. H. Kim et al. 2021), 15*S*‐ (J. Lee et al. 2020; Yi et al. 2020), and 15*R*‐LOXs (J. Lee et al. 2024a); however, such conversions of EPA for 18*R*‐ and 18*S*‐LOX have not been reported.

DiHEPEs as SPMs were synthesized from C20‐ and C22‐PUFAs via sequential reactions of two different positional‐selective single‐oxygenating LOXs. Specifically, RvE2 and 18*S*‐RvE2 can be synthesized from EPA via 18*R*‐ and 18*S*‐HEPE intermediates via a sequential reaction involving 18*R*‐ or 18*S*‐LOX, followed by 5*S*‐LOX, respectively. However, the production of these compounds has been hindered by the lack of identified 18‐LOXs. Thus, to achieve higher concentrations of RvE2 and 18*S*‐RvE2, the discovery or development of highly efficient 18‐LOXs is necessary.

**Figure 1:**
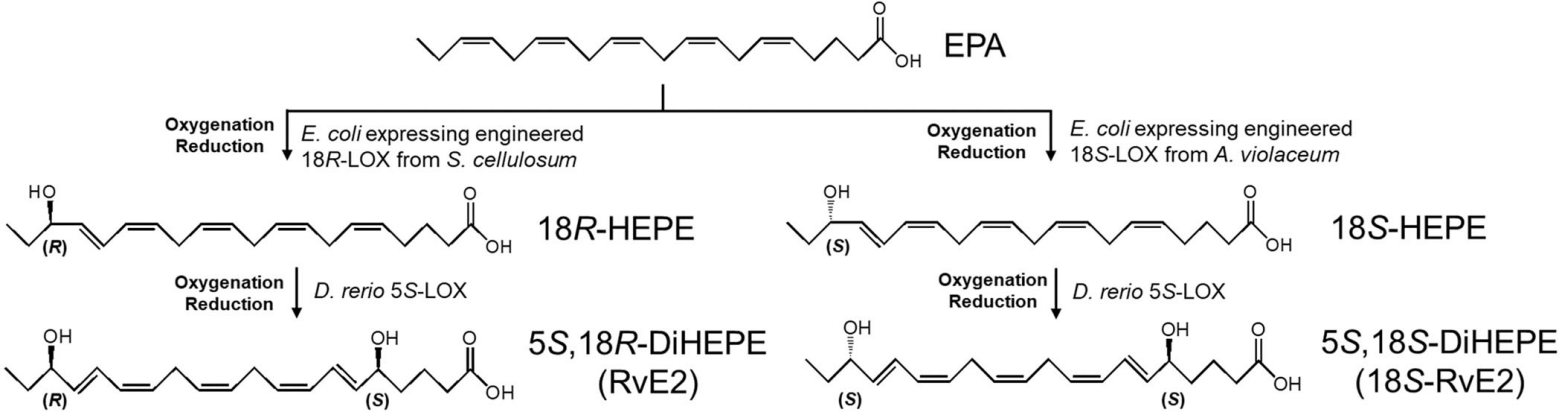
Biosynthetic pathway from EPA to 5*S*,18*R*‐DiHEPE (RvE2) and 5*S*,18*S*‐DiHEPE (18*S*‐RvE2) via 18*R*‐HEPE and 18*S*‐HEPE in the presence of cysteine as a reducing agent by the sequential reactions of the engineered 18*R*‐LOX from *S. cellulosum* or engineered 18*S*‐LOX from *A. violaceum* and *D. rerio* 5*S*‐LOX, respectively. The biosynthetic pathways from EPA to RvE2 and from 18*S*‐RvE2 via 5*S*‐HEPE are unavailable because the engineered 18*R*‐LOX and 18*S*‐LOX exhibited little activity for 5*S*‐HEPE.

In this study, 18*R*‐LOX (L423W/L424M/L568M variant of 15*R*‐LOX) and 18*S*‐LOX (L429W/L430M/L575M variant of 15*S*‐LOX) were developed from 15*R*‐LOX from *Sorangium cellulosum* (J. Lee et al. 2024a) and 15*S*‐LOX from *Archangium violaceum* (J. Lee et al. 2020), respectively, via structure‐guided enzyme engineering. By combining the engineered 18*R*‐ or 18*S*‐LOX with 5SLOX from *Danio rerio* (zebrafish), RvE2 and 18*S*‐RvE2 were quantitatively produced from EPA via 18*R*‐ and 18*S*‐HEPEs, respectively (Figure 1).

## 2 Materials and Methods

### 2.1 Materials

The EPA standard was purchased from Sigma‐Aldrich (St. Louis, MO, USA). The standards for 15*R*‐ and 15*S*‐hydroxyeicosatetraenoic acids (15*R*‐ and 15*S*‐HETEs), 15*S*‐ and 18(*S*/*R*)‐HEPEs, and RvE2 were obtained from Cayman Chemical (Ann Arbor, MI, USA). The SP825 absorbent resin was purchased from Ion Technology (Sungnam, Republic of Korea).

### 2.2 Preparation of HEPEs and DiHEPEs

15*R*‐ or 18*R*‐HEPE and 15*S*‐ or 18*S*‐HEPE were produced by *Escherichia coli* expressing 15*R*‐ or engineered 18*R*‐LOX from *S. cellulosum* and *E. coli* expressing 15*S*‐ or engineered 18*S*‐LOX from *A. violaceum*, respectively. The reactions were performed at 20°C in 50 mM 4‐(2‐hydroxyethyl)piperazine‐1‐ethane sulfornic acid (HEPES) buffer (pH 8.0) containing 2.0 g/L cells for LOXs from *S. cellulosum* or 1.0 g/L cells for LOXs from *A. violaceum*, 2.0 mM EPA, and 200 mM cysteine, as a reductant, with shaking at 200 rpm for 60 or 120 min, respectively. The optimal concentration of cysteine for the production of dihydroxy fatty acids was previously determined to be 200 mM (J. Lee et al. 2020). For the production of 5*S*,18*R*‐ and 5*S*,18*S*‐DiHEPEs, the reaction involving 5*S*‐LOX from *D. rerio* was performed at 20°C in 50 mM HEPES buffer (pH 8.0) containing 1.0 g/L (3.44 U/mL) enzyme, 0.5 mM 18*R*‐ or 18*S*‐HEPE, and 200 mM cysteine for 60 min, respectively.

After the reactions, the solutions were extracted with an equal volume of ethyl acetate, which was subsequently removed under reduced pressure using a rotary evaporator. The dried residue was dissolved in methanol. The reaction products, 18*R*‐ and 18*S*‐HEPEs, were purified as a single compound from the positional isomer mixture using a high‐performance liquid chromatography (HPLC) instrument (Agilent 1260, Santa Clara, USA) equipped with a chiral‐phase Lux Amylose‐1 column (Phenomenex, Torrance, USA) at 234 nm. The elution was performed at 30°C using acetonitrile supplemented with 3% methanol and 0.05% acetic acid, with a linear gradient of 27% acetonitrile and 63% acetonitrile at a flow rate of 0.5 mL/min for 45 min (J. Lee et al. 2024a).

The reaction products, 15*R*‐ and 15*S*‐HEPEs and 5*S*,18*R*‐ and 5*S*,18*S*‐DiHEPEs, were purified using HPLC equipped with a reversed‐phase Nucleosil C18 column (Phenomenex) at 202 nm. The elution was carried out at 30°C using a solvent system of acetonitrile containing 0.1% acetic acid. The gradient started at 50% acetonitrile for 5 min, followed by 100% acetonitrile for 5–22 min. The solvent composition was maintained at 100% acetonitrile for 22–27 min, then switched to a gradient 50% acetonitrile for 27–32 min, and maintained at 50% acetonitrile from 32 to 35 min at a flow rate of 0.25 mL/min (An et al. 2015). The collected fractions were treated with the SP825 adsorbent resin. After filtration to remove the resin, the solution was extracted using methanol. The extracts were used as HEPE and DiHEPE standards (Lee et al. 2020). Finally, 6*R*,8*R*‐, 8*R*,15*S*‐, and 7*S*,8*S*‐dihydroxyoctadecadienoic acids (DiHODEs) were prepared as described previously (Seo et al. 2019; Seo et al. 2016; Shin et al. 2016).

### 2.3 Microorganisms and Gene Cloning

The expression vector used in these experiments was the pET‐28a(+) plasmid, and the host strain was *Escherichia coli* C2566. The genes encoding the 15*R*‐ and 15*S*‐LOXs were sourced from *S. cellulosum* DSM14627 and *A. violaceum* DSM 52838, respectively. The cloning of the genes encoding 15*R*‐LOX from *S. cellulosum* (95.9% sequence identity to UniprotKB, S4XZS0), 15*S*‐LOX from *A. violaceum* (UniprotKB, A0A084ST31), and 5*S*‐LOX from *Danio rerio* (UniprotKB, F1R442) was performed as described previously (J. Lee et al. 2020; J. Lee et al. 2024a; Shin et al. 2023). Site‐directed mutagenesis was performed using a Quick‐Change kit (Stratagene, Beverly, MA, USA). The primers used for the 15*R*‐ and 15*S*‐LOX variants are listed in Supporting Information S1: Table S1. Each plasmid was transformed into *E. coli* C2566 via electroporation.

### 2.4 Cultivation Conditions

A colony of *E. coli* was inoculated into 5 mL of Luria–Bertani (LB) medium mixed with 0.1 mM kanamycin and cultivated at 37°C with shaking at 200 rpm overnight. The culture was transferred into a 2‐L‐baffled flask containing 1 L of LB medium supplemented with 0.1 mM kanamycin and further cultivated at 37°C with shaking at 200 rpm. When the optical density at 600 nm reached 0.7, enzyme expression was induced by adding 0.1 mM isopropyl‐β‐d‐thiogalactopyranoside. The culture was further incubated at 16°C overnight with shaking at 150 rpm, to promote high enzyme expression. The recombinant cells were harvested by centrifugation at 5000*g* at 4°C for 30 min. The harvested cells were washed twice with saline solution and used as biocatalysts for the production of HEPEs and DiHEPEs.

### 2.5 Enzyme Purification

The washed cells were resuspended in 50 mM phosphate buffer (pH 8.0) containing 10 mM imidazole and 300 mM NaCl and disrupted via sonication at 4°C for 30 min. The enzyme solution was recovered through centrifugation at 4°C for 20 min at 5000*g*. The cell‐free supernatant was obtained through filtration using a 0.45‐μm pore‐sized filter and subsequently applied to a fast protein liquid chromatography system (Bio‐Rad, CA, USA) equipped with a His‐Trap HP affinity column, which had been equilibrated with 50 mM phosphate buffer (pH 8.0) containing 300 mM NaCl. The protein purification protocol for chromatography purification included 300 mM NaCl to enhance purification efficiency (Sassenfeld and Brewer 1984). The high salt concentration aids in maintaining protein purity by reducing non‐specific binding. The bound protein was eluted using a buffer containing 250 mM imidazole and 300 mM NaCl at a flow rate of 1.5 mL/min. The active fractions were collected, concentrated, and used as the purified enzyme in subsequent experiments.

### 2.6 Enzyme Assay

One unit (U) of enzyme was defined as the amount of LOX required to generate 1 μmol of hydroperoxyeicosapentaenoic acid/min at 20°C and pH 8.0. The specific activities and kinetic parameters of 15*R*‐LOX and the engineered 18*R*‐LOX from *S. cellulosum*, as well as of 15*S*‐LOX and the engineered 18*S*‐LOX from *A. violaceum*, toward EPA were determined by measuring the absorbance at 234 nm using a spectrophotometer. The reactions were performed in 50 mM HEPES buffer (pH 8.0) for LOXs from *S. cellulosum* or in 50 mM HEPES buffer (pH 8.5) for LOXs from *A. violaceum* containing 1.0–6.0 μg/mL of purified enzyme and 10–800 μM EPA as the substrate for 3 min. The turnover rate (*k*_cat_, 1/min) and Michaelis–Menten constant (*K*_m_, mM) were calculated via nonlinear regression of the data using the GraphPad Prism software.

### 2.7 Optimization of pH and Temperature

The optimization of pH for enhanced production of 18*R*‐ or 18*S*‐HEPE from EPA by *E. coli* expressing engineered 18*R*‐ or 18*S*‐LOX, respectively, was evaluated at a constant temperature of 20°C. The pH was varied from 7.0 to 9.5 using 50 mM HEPES (pH 7.0–8.0), 3‐[4‐(2‐hydroxyethyl)piperazin‐1‐yl]propane‐1‐sulfonic acid (EPPS) (pH 7.5–8.5), and 2‐(Cyclohexylamino)ethane‐1‐sulfonic acid (CHES) (pH 8.5–9.5) buffers. The optimization of the temperature for the enhanced production of 18*R*‐ or 18*S*‐HEPE was investigated by varying the temperature from 15°C to 35°C in 50 mM HEPES (pH 7.5) or 50 mM EPPS (pH 8.5) buffer, respectively. The reactions were carried out using 1.0 mM EPA, 1.0 g/L cells, and 200 mM cysteine for 15 min.

### 2.8 Optimization of Substrate and Cell Concentrations

To optimize the substrate concentration for the enhanced production of 18*R*‐ or 18*S*‐HEPE, the reactions were performed at 20°C in 50 mM HEPES (pH 7.5) or 50 mM EPPS (pH 8.5) buffer, respectively, with 1.0 g/L cells and 200 mM cysteine by varying the EPA concentration from 0.5 to 5.0 mM for 30 min. The cell concentration was optimized by varying it from 0.5 to 8.0 g/L using 4.0 mM EPA or from 0.5 to 8.0 g/L using 3.0 mM EPA. For the enhanced production of RvE2 or 18*S*‐RvE2, the reactions were performed at 20°C in 50 mM HEPES buffer (pH 8.0) containing 1.0 g/L of 5*S*‐LOX from *D. rerio*, 0.1 mM Zn^2+^, and 200 mM cysteine by varying the concentration of 18*R*‐ or 18*S*‐HEPE from 0.1 to 1.0 mM for 60 min, respectively.

### 2.9 Homology Modeling and Substrate Docking

Homology models of 15*R*‐LOX from *S. cellulosum* and 15*S*‐LOX from *A. violaceum* were constructed using the structures of linoleate 11*R*‐LOX from *Cyanothece* sp. (protein data bank, PDB, 5MED) and arachidonate 15*S*‐LOX from *P. aeruginosa* (PDB, 5IR4), which exhibited sequence identities of 45.4% and 50.4%, respectively. The modeling was performed using the Discovery Studio software (San Diego, CA, USA), as described previously (J. Lee et al. 2020; J. Lee et al. 2024a). To investigate substrate binding, the EPA substrate was docked into the active sites of these homology models using the C‐DOCKER module, with docking poses generated by simulated annealing and random rigid‐body rotations. The energy minimization of the resulting protein–ligand complex structures was carried out using the CHARMM force field.

### 2.10 Determination of Specific Rotations

HEPE samples were derived from the conversion of EPA using the engineered 18*R*‐LOX from *S. cellulosum* and 18*S*‐LOX from *A. violaceum*, and the DiHEPE samples were produced from 18*R*‐ and 18*S*‐HEPEs using 5*S*‐LOX from *D. rerio*. The specific rotations of the samples were measured at 20°C using a polarimeter at a wavelength of 589 nm in a concentration range of 0.05–0.23 mg/mL (J. Lee et al. 2020; J. Lee et al. 2024a). Control samples for the *R‐* and *S‐*forms, as well as the *R*,*R*‐, *R*,*S*‐, and *S*,*S*‐forms, were represented by the 15*R*‐ and 15*S*‐HETEs and the 8*R*,15*S*‐ and 7*S*,8*S*‐DiHODEs, respectively.

### 2.11 Analytical Methods

The concentrations of the products were determined using the calibration curves of the product standards (Supporting Information S1: Table S2) and an HPLC instrument equipped with a Nucleosil C18 column. A chirality analysis was performed using a Lux Amylose‐1 column. The analytical conditions were consistent with those described in the section on the preparation of HEPEs and DiHEPEs.

A liquid chromatography–tandem mass spectrometry (LC‐MS/MS) analysis of 18*R*‐ and 18*S*‐HEPEs, as well as 5*S*,18*R*‐ and 5*S*,18*R*‐DiHEPEs, was performed using a Thermo‐Finnigan LCQ Deca XP Plus ion trap mass spectrometer (Thermo Fisher Scientific, Waltham, MA, USA) at the National Instrumentation Center of Seoul National University (Seoul, Republic of Korea). The samples were ionized using electrospray ionization under the following conditions: capillary temperature, 300°C; ion source voltage, 2.7 kV; capillary voltage in the negative mode, 10 V; capillary voltage in the positive mode, 46 V; collision energy, 35 eV; tube lens voltage, 100 V; average scan time, 0.01 min; polarity switching time, 0.02 min; and abundance for the precursor ions at the specified collision energy, 35%.

## 3 Results

### 3.1 Alteration of 15‐LOXs to 18‐LOXs for EPA via Structure‐Guided Enzyme Engineering

To identify the key positional‐determinant residues of 15*R*‐LOX from *S. cellulosum*, EPA was docked into the substrate‐binding pocket. The residues that frequently interacted with EPA in the docking model were identified as Leu423, Leu424, Leu568, and Met603 (Supporting Information S1: Figure S1), corresponding to Leu439, Leu430, Leu575, and Thr610 in 15*S*‐LOX from *A. violaceum*, based on a sequence alignment (Supporting Information S1: Figure S2).

**Figure 2:**
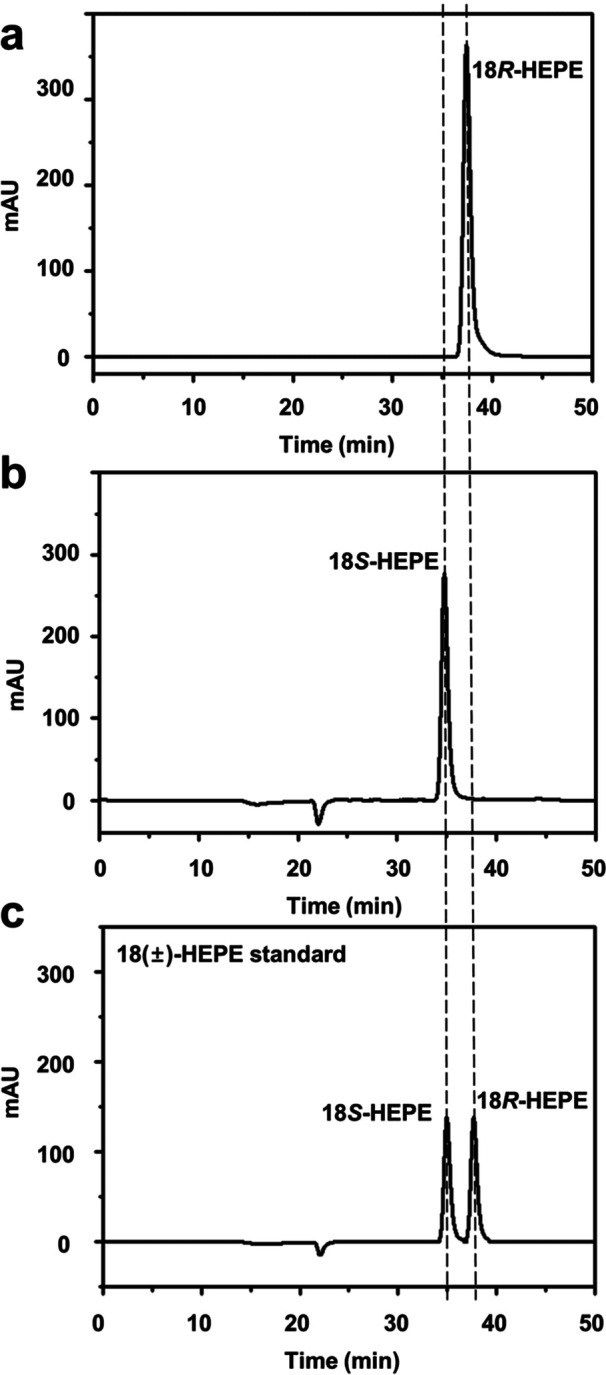
Chiral‐phase HPLC chromatograms of the products obtained from the conversion of EPA using engineered 18*R*‐ and 18*S*‐LOXs with an 18(±)‐HEPE standard. (a) HPLC chromatograms of the product obtained from the conversion of EPA by engineered 18*R*‐LOX from *S. cellulosum* in the presence of cysteine. (b) HPLC chromatograms of the product obtained from the conversion of EPA by engineered 18*S*‐LOX from *A. violaceum* in the presence of cysteine. (c) HPLC chromatograms of 18(±)‐HEPE standard. The *R‐* and *S*‐forms were confirmed using a polarimeter. The reactions were performed at 25°C in 50 mM HEPES buffer (pH 8.0) containing 1.0 mM EPA, 1.0 g/L enzyme, and 100 mM cysteine for 20 min.

Mutations of 15*R*‐LOX from *S. cellulosum* were performed at positions 423, 424, 568, and 603 by substituting these residues with bulkier amino acids. The single‐site variants with charged amino acids (Asp, Lys, and His) at position 423 or 424 exhibited no detectable enzyme activity, whereas those with neutral amino acids (Met, Phe, Tyr, and Trp) showed activity (Table 1). The wild‐type 15*R*‐LOX and engineered 18*R*‐LOX from *S. cellulosum* converted EPA into 15*R*‐HEPE without producing 15*S*‐HEPE (J. Lee et al. 2024a) and into 18*R*‐HEPE without generating 18*S*‐HEPE, respectively (Figure 2a). Similarly, the wild‐type 15*S*‐LOX and engineered 18*S*‐LOX from *A. violaceum* converted EPA into 15*S*‐HEPE without producing 15*R*‐HEPE (J. Lee et al. 2020) and into 18*R*‐HEPE without generating 18*S*‐HEPE, respectively (Figure 2b). Therefore, chirality was not assigned. The L423W, L423W/L424M, and L423W/L424M/L568M variants of 15*R*‐LOX from *S. cellulosum* produced 36.1%, 51.4%, and 72.5% 18*R*‐HEPE relative to 15*R*‐ and 18*R*‐HEPEs as the single‐, double‐, and triple‐site variants with the highest proportion of 18*R*‐HEPE, respectively, whereas the L430W, L429W/L430M, and L429W/L430M/L575M variants of 15*S*‐LOX from *A. violaceum* produced 51.5%, 60.7%, and 81.8% 18*S*‐HEPE relative to 15*S*‐ and 18*S*‐HEPEs, respectively. However, the M609W variation in 15*R*‐LOX from *S. cellulosum* did not significantly affect the positional selectivity. Therefore, the L423W/L424M/L568M and L429W/L430M/L575M variants were considered as being engineered 18*R*‐ and 18*S*‐LOXs, respectively.

**Table 1:** Specific activities and HEPE positional isomer ratios of wild‐type and variant LOXs toward EPA.

LOX	Residues	MW of two residues^a^	Specific activity (U/g)	Positional isomer ratio (%)	
				18‐HEPE	15‐HEPE
*S. cellulosum* 15*R*‐LOX	Wild‐type	262	206 ± 2.5	0.00	100
	L423D	264	ND		
	L423K	277	ND		
	L423H	286	ND		
	L423W	335	108 ± 2.2	36.1	64.9
	L424D	264	ND		
	L424K	277	ND		
	L424H	286	ND		
	L424M	280	180 ± 2.8	7.30	92.7
	L424F	296	158 ± 1.4	13.6	86.4
	L424Y	312	139 ± 2.0	21.9	78.1
	L424W	335	115 ± 1.7	33.4	66.6
	L568W		108 ± 2.3	17.9	82.1
	M603W		170 ± 1.6	0.20	99.8
	L423Y/L424F	346	86.8 ± 0.8	35.9	64.1
	L423M/L424W	353	69.5 ± 0.4	47.8	52.2
	L423W/L424M	353	61.7 ± 0.7	51.4	48.6
	L423Y/L424Y	362	ND		
	L423W/L424F	369	ND		
	L423W/L424W	408	ND		
	L423W/L424M/L568V	353	32.2 ± 0.3	55.9	45.1
	L423W/L424M/L568M	353	20.5 ± 0.5	72.5	27.5
	L423W/L424M/L568F	353	ND		
*A. violaceum* 15*S*‐LOX	Wild‐type	262	185 ± 3.1	ND	100
	L429W	335	127 ± 3.1	50.8	49.2
	L430W	335	110 ± 1.5	51.5	48.5
	L429W/L430M	353	70.8 ± 2.6	60.7	39.3
	L429W/L430M/L575M	353	22.7 ± 0.9	81.8	18.2

^a^ Molecular weight (MW) of two amino acid residues at positions 423 and 424 of 15*R*‐LOX from *S. cellulosum*.

The specific activities and kinetic parameters of the wild‐type LOXs and single‐, double‐, and triple‐site variants were determined (Table 2). The specific activity and catalytic efficiency (*k*_*cat*_ */K*_*m*_) followed the order: wild‐type LOXs > single‐site variants > double‐site variants > triple‐site variants, whereas the positional isomer ratios of the 18R‐ and 18*S*‐HEPEs followed the opposite order: triple‐site variants > double‐site variants > single‐site variants > wild‐type LOXs.

**Table 2:** Specific activities and kinetic parameters of wild‐type and variant 15‐LOXs.

Organisms	Product	Type	Specific activity (μmol/min/mg)	*K*_m_(μmol/L)	*k*_*cat*_(1/s)	*k*_*cat*_ */K*_m_ (L/s/μmol)
*Sorangium cellulosum*	15*R*‐HpEPE^a^	Wild‐type	38.4 ± 0.15	41.9	68.0	1.62
	15*R*‐and 18*R*‐HpEPEs	L423W	20.1 ± 0.01	69.8	104.8	1.50
		L423W/L424M	11.7 ± 0.14	43.3	22.7	0.52
		L423W/L424M/L568M	3.04 ± 0.03	253.7	90.8	0.36
*Archangium violaceum*	15*S*‐HpEPE	Wild‐type	23.3 ± 0.21	40.7	61.4	1.51
	15*S*‐and 18*S*‐HpEPEs	L429W	16.7 ± 0.17	35.6	51.8	1.45
		L429W/L430M	8.06 ± 0.08	57.2	48.7	0.85
		L429W/L430M/L575M	4.31 ± 0.05	221.3	117.5	0.53

^a^ HpEPE: hydroperoxyeicosapentaenoic acid.

### 3.2 Identification of the Products Obtained From the Conversion of EPA by Engineered 18*R*‐ and 18*S*‐LOXs

The LC‐MS/MS spectra of the products obtained from the conversion of EPA by engineered 18*R*‐ and 18*S*‐LOXs are reported in Supporting Information S1: Figure S3. Key fragment peaks were observed at a mass‐to‐charge ratio (*m*/*z*) of 259.1, resulting from cleavage at the C18 position, where one hydroxyl group was attached to the EPA, indicating that the products were 18‐HEPEs. A comparison of the retention times of the products with that of an authentic 18(±)‐HEPE standard in the chiral‐HPLC revealed differences between the products derived from engineered 18*R*‐ and 18*S*‐LOXs (Figure 2).

The specific rotations of the 15*R*‐ and 15*S*‐HETE standards, which served as controls for the *R*‐ and *S*‐forms, were ${\left[\alpha \right]}_{D}^{20}$ = −3.5 (0.23 g/L) and +6.2 (0.07 g/L), respectively. The specific rotations of the 18‐HEPEs produced from EPA by the engineered 18*R*‐ and 18*S*‐LOXs were −2.9 (0.10 g/L) and +5.6 (0.11 g/L), respectively, indicating that the products were 18*R*‐ and 18*S*‐HEPEs.

### 3.3 Identification of the Products Obtained From the Conversion of 18*R*‐ and 18*S*‐HEPEs Using 5*S*‐LOX From *D. Rerio*

**Figure 3:**
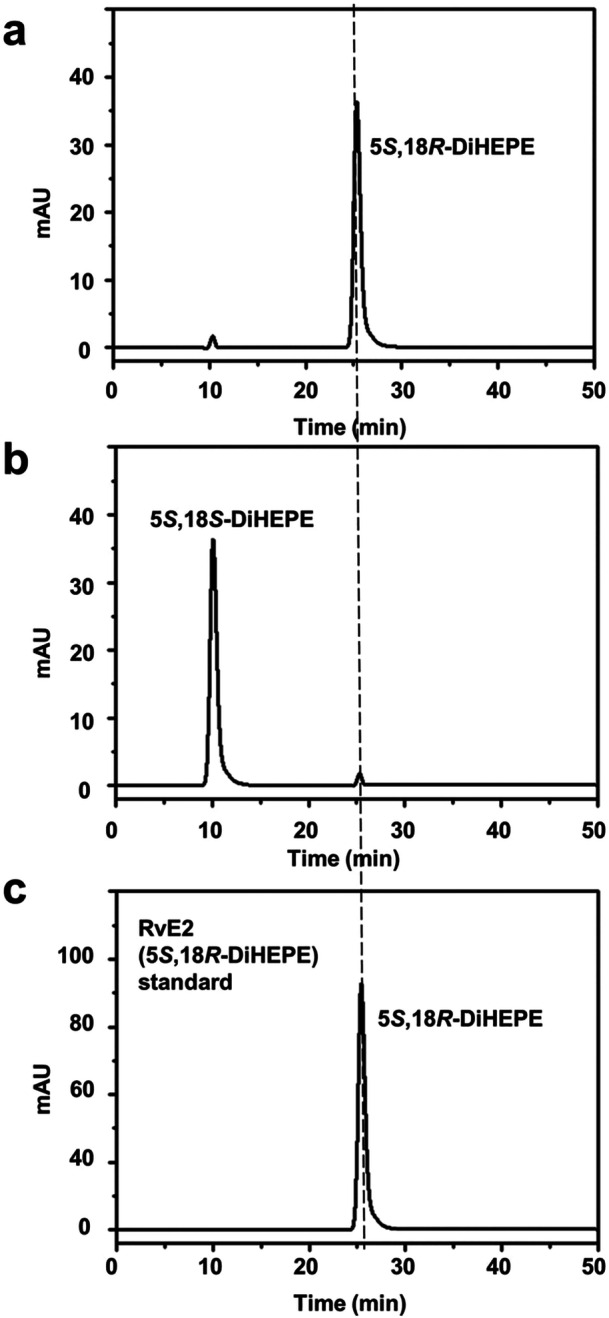
Chiral‐phase HPLC chromatograms of the products obtained from the conversion of 18*R*‐ and 18*S*‐HEPEs by 5*S*‐LOX from *D. rerio* using the RvE2 (5*S*,18*R*‐DiHEPE) standard. (a) HPLC chromatogram of the product obtained from the conversion of 18*R*‐HEPE using *D. rerio* 5*S*‐LOX in the presence of cysteine. (b) HPLC chromatogram of the product obtained from the conversion of 18*S*‐HEPE by 5*S*‐LOX from *D. rerio* in the presence of cysteine. (c) HPLC chromatogram of the RvE2 standard. The substrates 18*R*‐ and 18*S*‐HEPEs were obtained from the conversion of EPA by engineered 18*R*‐LOX from *S. cellulosum* and engineered 18*S*‐LOX from *A. violaceum*, respectively. The reactions were performed at 20°C in 50 mM HEPES buffer (pH 8.0) containing 0.5 mM 18‐HEPE, 1.0 g/L enzyme, and 100 mM cysteine for 60 min.

Key fragment peaks in the LC‐MS/MS spectra of the products obtained from the conversion of 18*R*‐ and 18*S*‐HEPEs using 5*S*‐LOX from *D. rerio* were observed at *m*/*z* 217.4 and 275.1, respectively (Supporting Information S1: Figure S4). These mass‐to‐charge ratios corresponded to cleavages at the C5 and C18 positions at which two hydroxyl groups were attached to EPA, respectively. Based on these *m*/*z* values, the products were identified as 5,18‐DiHEPEs. The 5,18‐DiHEPE product obtained from the conversion of 18*R*‐HEPE by 5*S*‐LOX from *D. rerio* (Figure 3a) exhibited the same retention time as the RvE2 (5*S*,18*R*‐DiHEPE) standard in the chiral‐HPLC (Figure 3c), indicating that the product is 5*S*,18*R*‐DiHEPE. Its enantiomeric excess was calculated as 95.6% based on major and minor peaks observed in the chiral‐HPLC shown in in Figure 3a. In contrast, the 5,18‐DiHEPE product obtained from the conversion of 18*S*‐HEPE by 5*S*‐LOX (Figure 3b) showed a different retention time, confirming a stereochemical distinction. This product was identified primarily as 5*S*,18*S*‐DiHEPE, with an enantiomeric excess of 96.4% based on peak areas in the chiral‐HPLC in Figure 3b.

The specific rotations of the 6*R*,8*R*‐, 8*R*,15*S*‐, and 7*S*,8*S*‐DiHODEs, which served as controls for the *R*,*R*‐, *S*,*R*‐, and *S*,*S*‐forms, were ${\left[\alpha \right]}_{D}^{20}$ = −15.0 (0.11 g/L), −0.08 (0.13 g/L), and +16.8 (0.20 g/L), respectively. The specific rotation of the 5,18‐DiHEPE produced from 18*R*‐HEPE using 5*S*‐LOX was −0.10 (0.06 g/L), which was close to zero, confirming that the product is 5*S*,18*R*‐DiHEPE. The specific rotation of the 5,18‐DiHEPE produced from 18*S*‐HEPE was +48.2 (0.05 g/L), indicating that the product is 5*S*,18*S*‐DiHEPE. These results provide reliable structural identification. Therefore, further structural analysis, such as NMR, is not essential for product determination.

### 3.4 Production of 18*R*‐ or 18*S*‐HEPE From EPA by *E*. *Coli* Expressing 18*R*‐ or 18*S*‐LOX

The production of 18*R*‐HEPE from EPA by *E. coli* expressing engineered 18*R*‐LOX from *S. cellulosum* was maximal at pH 7.5°C and 20°C (Supporting Information S1: Figure S5), whereas the production of 18*S*‐HEPE by *E. coli* expressing engineered 18*S*‐LOX from *A. violaceum* was maximal at pH 8.5°C and 20°C (Supporting Information S1: Figure S6). The optimal substrate and cell concentrations for maximal production were 4.0 mM EPA and 2.0 g/L cells for producing 18*R*‐HEPE (Supporting Information S1: Figure S7) and 3.0 mM EPA and 2.0 g/L cells for producing 18*S*‐HEPE (Supporting Information S1: Figure S8).

**Figure 4:**
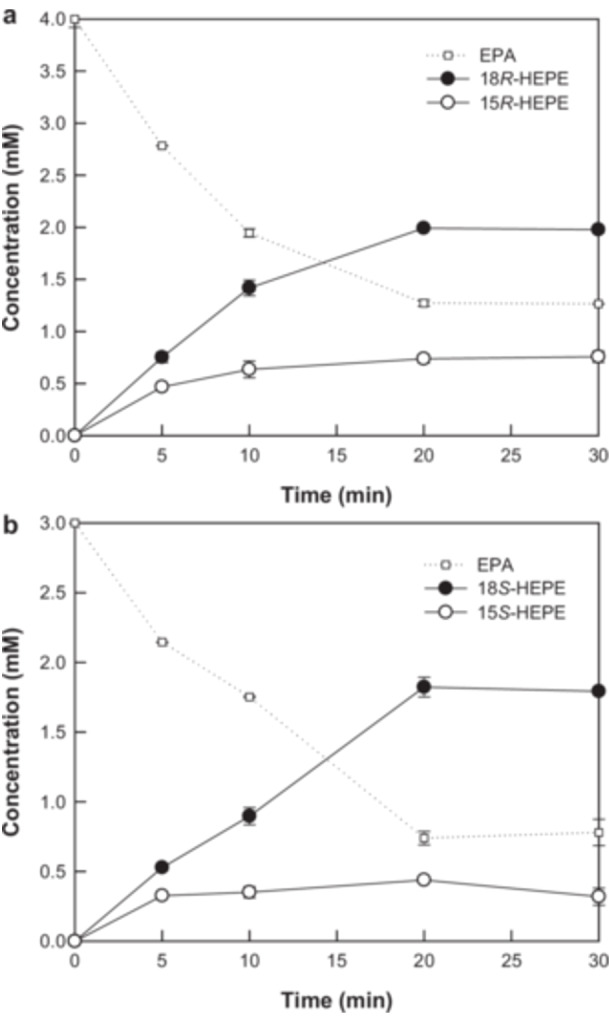
Biotransformation of EPA into 18*R*‐ and 18*S*‐HEPE by *E. coli* expressing engineered 18*R*‐LOX from *S. cellulosum* and engineered 18*S*‐LOX from *A. violaceum*. (a) Biotransformation of EPA into 18*R*‐HEPE by *E. coli* expressing engineered 18*R*‐LOX from *S. cellulosum*. (b) Biotransformation of EPA into 18*S*‐HEPE by *E. coli* expressing engineered 18*S*‐LOX from *A. violaceum*. The symbols indicate EPA (□), 18‐HEPEs (●), and 15‐HEPEs (○). The error bars and data points indicate the standard deviation and means of the three experiments, respectively.

Using the optimized conditions, *E. coli* expressing engineered 18*R*‐ or 18*S*‐LOX converted 4.0 mM (1218 mg/L) or 3.0 mM (914 mg/L) EPA into 2.0 mM (641 mg/L) 18*R*‐HEPE or 1.8 mM (577 mg/L) 18*S*‐HEPE, respectively, after 20 min (Figure 4). The positional ratio of 18*R*‐HEPE relative to 15*R*‐ and 18*R*‐HEPEs or 18*S*‐HEPE relative to 15*S*‐ and 18*S*‐HEPEs was 73.0% or 80.6% at 20 min because of the production of the byproduct, i.e., 0.76 mM 15*R*‐HEPE or 0.44 mM 15*S*‐HEPE, respectively.

### 3.5 Production of 5*S*,18*R*‐ or 5*S*,18*S*‐DiHEPE From 18*R*‐ or 18*S*‐HEPE by *D. Rerio* 5*S*‐LOX

**Figure 5:**
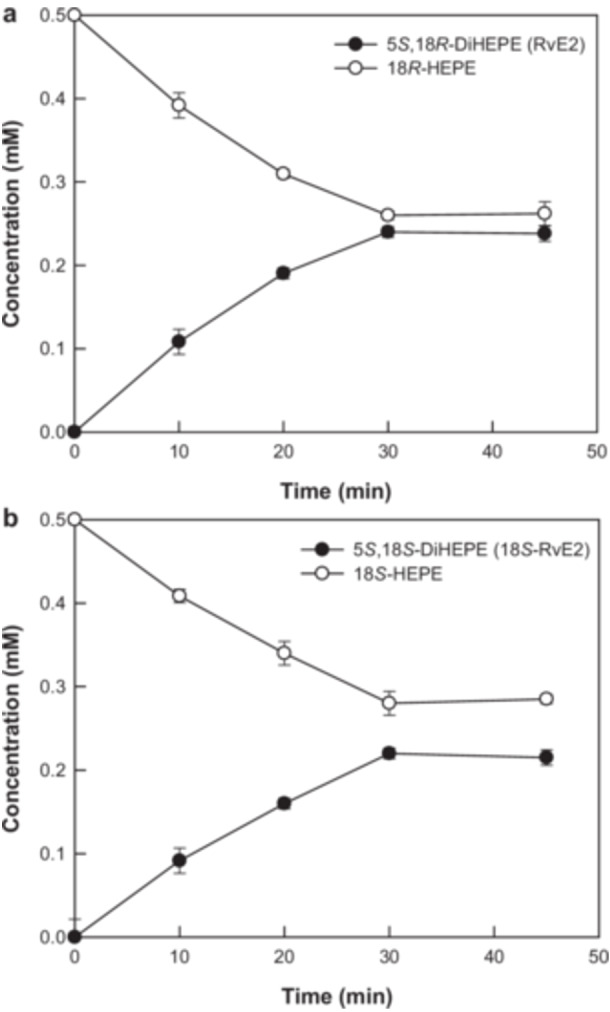
Biotransformation of 18*R*‐ and 18*S*‐HEPE into 5*S*,18*R*‐DiHEPE (RvE2) and 5*S*,18*S*‐DiHEPE (18*S*‐RvE2) of 5*S*‐LOX from *D. rerio*. (a) Biotransformation of 18*R*‐HEPE into RvE2. (b) Biotransformation of 18*S*‐HEPE into 18*S*‐RvE2. The purified 18*R*‐ or 18*S*‐HEPE was obtained from the conversion of EPA by *E. coli* expressing engineered 18*R*‐LOX from *S. cellulosum* or engineered 18*S*‐LOX from *A. violaceum*, respectively, and was used as a substrate. The symbols indicate 18‐HEPEs (○) and 5,18‐DiHEPEs (●). The error bars and data points indicate the standard deviation and means of the three experiments, respectively.

The optimal substrate concentration for the enhanced production of RvE2 or 18*S*‐RvE2 from 18*R*‐ or 18*S*‐HEPE as a substrate by 5*S*‐LOX from *D. rerio* was determined to be 0.5 mM (Supporting Information S1: Figure S9). Based on the conditions used for the production of 5‐HEPE from EPA by 5*S*‐LOX (Shin et al. 2023), the conditions for the production of 5*S*,18*R‐*DiHEPE (RvE2) or 5*S*,18*S*‐DiHEPE (18*S*‐RvE2) from 18*R*‐ or 18*S*‐HEPE were set to 20°C, pH 8.0, 0.1 mM Zn^2+^, 1.0 g/L enzyme, and 0.5 mM 18*R*‐ or 18*S*‐HEPE. Under these conditions, 5*S*‐LOX converted 0.5 mM (160 mg/L) 18*R*‐ or 18*S*‐HEPE into 0.24 mM (81 mg/L) RvE2 or 0.22 mM (74 mg/L) 18*S*‐RvE2 in 30 min, respectively (Figure 5).

## 4 Discussion

The residues that frequently interacted with EPA in the docking model were identified as Leu423, Leu424, Leu568, and Met603 in 15*R*‐LOX from *S. cellulosum* (Supporting Information S1: Figure S1). Although Met603 was not a positional‐determinant residue, Leu423, Leu424, and Leu568 were determinant residues, corresponding to Leu439, Leu430, and Leu575 in 15*S*‐LOX from *A. violaceum* (Supporting Information S1: Figure S2). The wild‐type 15*S*‐LOX from *A. violaceum* produces 100% 5,15‐dihydroxyeicosatetraenoic acid (5,15‐DiHETE), whereas the L429A/L430A variant with smaller residues produces 98% 5,12‐DiHETE (J. Lee et al. 2020). These results suggest that substituting these residues with bulkier residues can shift the positional selectivity from 15‐LOX activity to 18‐LOX activity.

To generate a variant with the highest positional isomer ratio of 18*R*‐HEPE by mutations of 15*R*‐LOX from *S. cellulosum*, key positional‐determinant residues at positions 423, 424, and 568 were substituted with bulkier amino acids. The positional isomer ratio of 18*R*‐HEPE to relative to 15*R*‐ and 18*R*‐HEPEs increased with the size of the neutral amino acid at position 424 in 15*R*‐LOX, following the order: Leu (molecular weight [MW] = 131) in wild‐type 15*R*‐LOX < Met (MW = 149) < Phe (MW = 165) < Tyr (MW = 181) < Trp (MW = 204) (Table 1). As the size of the neutral amino acids at positions 423 and 424 increased up to a combined MW of 353 Da, the positional isomer ratio of 18*R*‐HEPE followed the order: L423/L424 (MW = 262) in wild‐type 15*R*‐LOX < L423Y/L424F (MW = 346) < L423W/L424M (MW = 353). However, the double‐site variants with MWs exceeding 353 Da exhibited no detectable activity, possibly due to steric hindrance from the bulky amino acids.

Similarly, as the size of the neutral amino acids at position 568 in the L423W/L424M variant increased from Leu to Tyr, the positional isomer ratio of 18*R*‐HEPE followed the order: Leu in wild‐type 15*R*‐LOX < Val (MW = 117) < Met. However, there was no detectable activity when a large residue such as Phe or Tyr was present at position 568, which is likely due to steric hindrance caused by the large amino acids. The optimal size of the neutral amino acid at position 568 in the L423W/L424M variant was determined to be Met, as observed in the L423W/L424M/L568M variant. Therefore, the L429W/L430M/L575M variant of 15*S*‐LOX and the corresponding L423W/L424M/L568M variant of 15*R*‐LOX were considered to be 18*R*‐ and 18*S*‐LOXs, respectively. Furthermore, the size of the three residues at the bottom of the active‐site pocket appeared to be a key determinant of the positional selectivity of the LOXs.

**Figure 6:**
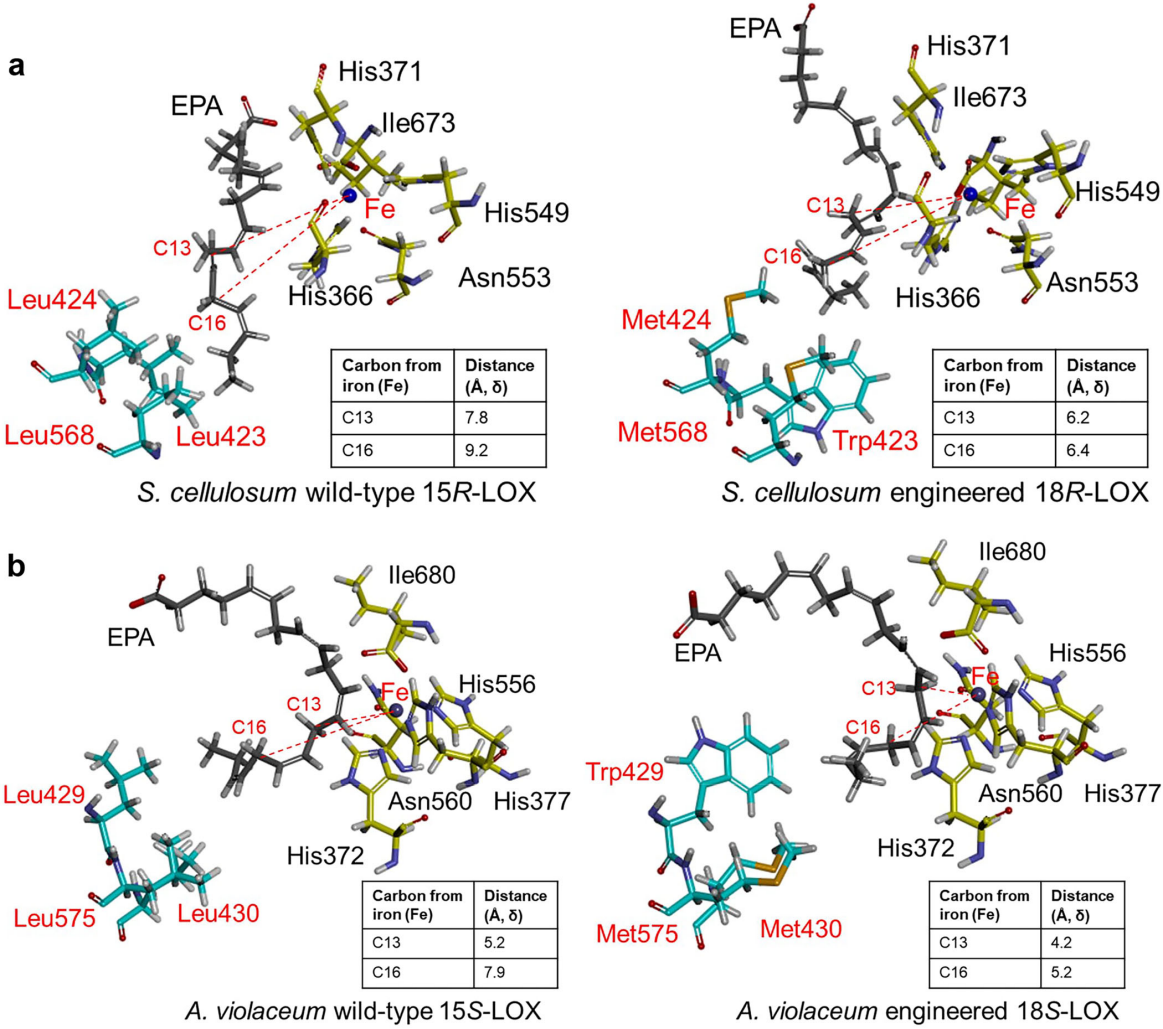
Docking models of EPA as a substrate in the substrate‐binding pockets of both wild‐type and engineered LOXs. (a) Docking models of EPA for wild‐type 15*R*‐LOX and engineered 18*R*‐LOX from *S. cellulosum*. (b) Docking models of EPA for wild‐type 15*S*‐LOX and engineered 18*S*‐LOX from *A. violaceum*. The metal‐binding residues were His366, His373, His549, Asn553, and Ile673 in LOX from *S. cellulosum* and His372, His377, His 556, Asn560, and Ile680 in LOX from *A. violaceum*. The red residues significantly contributed to regiospecificity.

Molecular‐docking models revealed that three Leu in the wild‐type 15‐LOXs were replaced by two Met and one Trp in the engineered 18‐LOXs, which were located at the bottom of the active‐site pocket (Figure 6). The engineered LOXs carrying bulkier residues in the active‐site pocket allowed EPA to penetrate more shallowly into the substrate‐binding pocket; thus, reducing the distance between the Fe ion and the C16 position in EPA and facilitating oxygenation at the C18 position. Consequently, the engineered LOXs exhibited the activities of 18‐LOXs rather than those of 15‐LOXs.

18*R*‐ and 18*S*‐HEPEs were produced from EPA using recombinant cells as they are more stable than the enzyme as a biocatalyst for the production of 15*R*‐ and 15*S*‐HEPEs (J. Lee et al. 2020; J. Lee et al. 2024a). Conversely, 5*S*‐LOX from *D. rerio* has been used in the biotransformation of PUFAs using purified enzyme rather than recombinant cells, as the formed hydroxy fatty acids are rapidly decreased by the cells (Shin et al. 2023). In this study, 5*S*‐LOX from *D. rerio* was used to produce RvE2 and 18*S*‐RvE2 from 18*R*‐ and 18*S*‐HEPEs, respectively, because 18‐LOXs exhibited a too‐low activity toward 5*S*‐HEPE as a substrate, which was produced from EPA using 5*S*‐LOX. Thus, RvE2 or 18*S*‐RvE2 was produced from EPA by sequential reactions using *E. coli* expressing engineered 18*R*‐ or 18*S*‐LOX, respectively, followed by purified 5*S*‐LOX (Figure 1).

*E. coli* expressing engineered 18*R*‐ or 18*S*‐LOX produced 2.0 mM (641 mg/L) 18*R*‐HEPE or 1.8 mM (577 mg/L) 18*S*‐HEPE from EPA (Figure 4), which was 189,000‐ or 721,000‐fold higher than the amounts (3.4 ng/mL 18*R*‐HEPE or 0.8 ng/mL 18*S*‐HEPE, respectively) produced by acetylated COX‐2 (Oh et al. 2011). These results demonstrate the high efficiency of the engineered 18‐LOXs for the production of 18‐HEPEs. Although RvE2 and 18*S*‐RvE2 have been previously identified (Oh et al. 2011), this study represents the first qualitative production of RvE2 and 18*S*‐RvE2 (Figure 5). RvE2 has been shown to inhibit chemoattractant‐stimulated polymorphonuclear leukocyte recruitment, resolve peritonitis, and alleviate depression (Serhan et al. 2022); however, the pharmacological efficacy of 18*S*‐RvE2 remains unreported. Therefore, the biotechnological production of these SPMs is significant for pharmacological studies because they can be prepared in greater quantities.

## 5 Conclusions

In conclusion, mutagenesis of the amino acids selected based on the analysis of favorable interactions at the active site resulted in the creation of the L423W/L424M/L568M variant of 15*R*‐LOX from *S. cellulosum* and the L429W/L430M/L575M variant of 15*S*‐LOX from *A. violaceum*, which were identified as 18*R*‐ and 18*S*‐LOXs, respectively. These engineered LOXs produced the highest concentrations of 18*R*‐ and 18*S*‐HEPEs ever reported. Furthermore, RvE2 and 18*S*‐RvE2 were qualitatively produced from the conversion of the prepared 18*R*‐ and 18*S*‐HEPEs by 5*S*‐LOX from *D. rerio*, respectively. To the best of our knowledge, this is the first qualitative production of RvE2 and 18*S*‐RvE2. Our findings may facilitate the efficient production of RvE2 and 18*S*‐RvE2, advancing studies on their pharmacological activities.

## Conflicts of Interest

The authors declare no conflicts of interest.

## Supporting information

18‐LOX_SI_ver3.

## Data Availability

The data that support the findings of this study are available from the corresponding author upon reasonable request.
