# Supplementary material for: Altering 15‐Lipoxygenases to 18‐Lipoxygenases and Their Application to the Production of 5,18‐Dihydroxyeicosapentaenoic Acids
Source: Biotechnol Bioeng. 2025 Apr 16;122(7):1759–69. doi: 10.1002/bit.28995 (PMC12152508; doi:10.1002/bit.28995)
Supplement: Supplementary file 1 — 18‐LOX_SI_ver3. [file BIT-122-1759-s001.docx]

**Supporting Information**

**Supplement Figures**


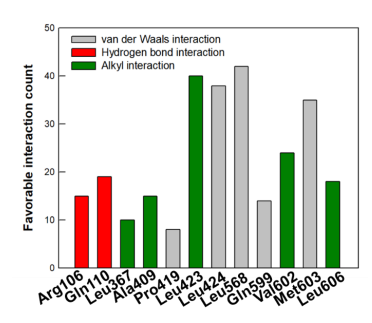


**Figure S1.** Favorable interaction counts between amino acid residues in *Sorangium cellulosum* 15*R*-lipoxygenase (LOX) and eicosapentaenoic acid (EPA) in 50 docking models. The red, green, and gray bars indicate hydrogen bond, alkyl, and van der Waals interactions, respectively. The residues exhibiting more than 30 favorable interaction counts were selected as Leu423, Leu424, Leu568, and Met603.


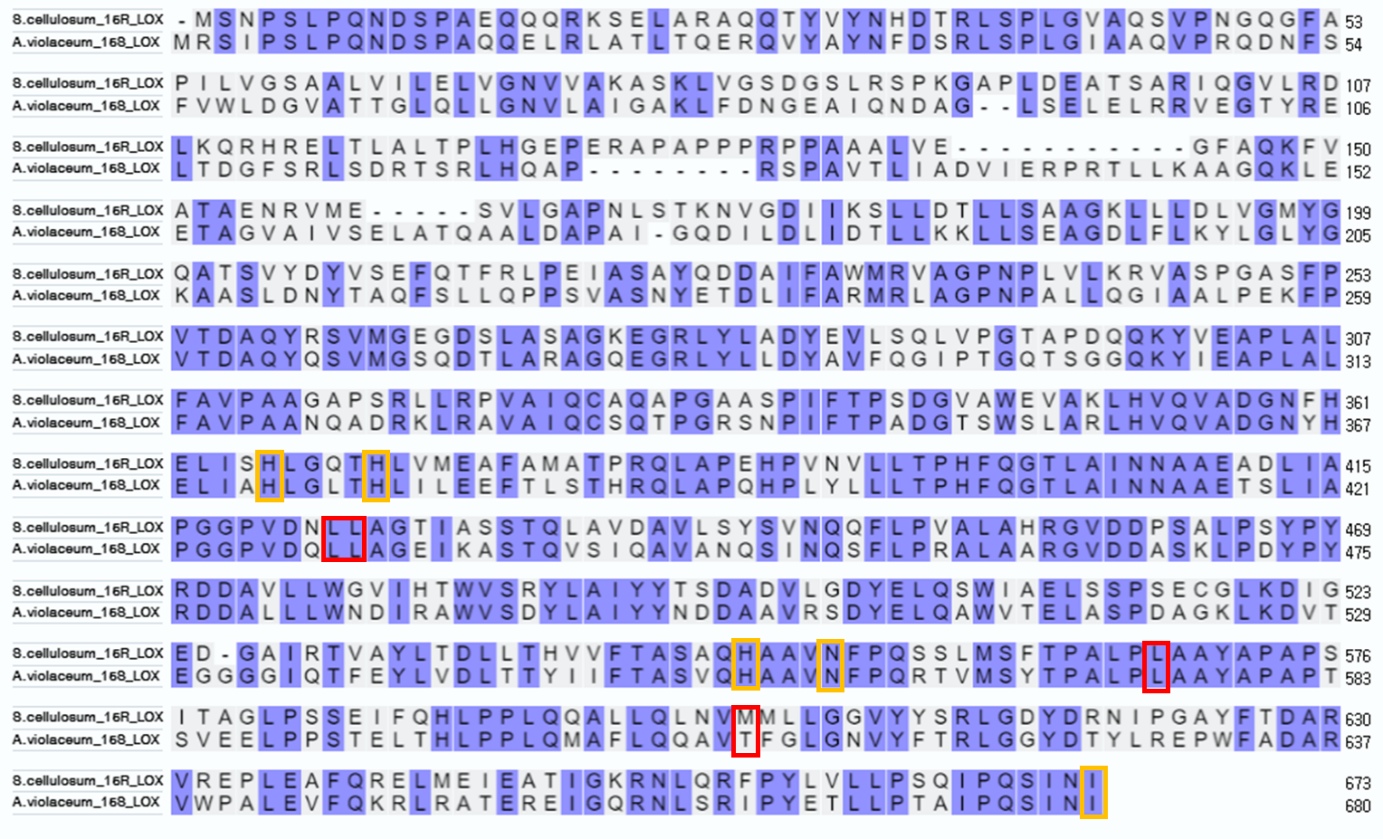


**Figure S2.** Alignment of amino acid sequences of *S. cellulosum* 15*R*-LOX and *Archangium violaceum* 15*S*-LOX. The metal-binding residues are completely conserved. These include three histidine residues (His366, His371, and His549 of *S. cellulosum* 15*R*-LOX and His372, His377, and His556 of *A. violaceum* 15*S*-LOX) and the other two residues (Asn553 and Ile673 of *S. cellulosum* 15*R*-LOX and Asn560 and Ile680 of *A. violaceum* 15*S*-LOX). The orange boxes indicate metal-binding residues (catalytic residues). Red boxes indicate the selected residues proposed as candidate regiospecific determinants: Leu423, Leu424, Leu568, and Met603 of *S. cellulosum* 15*R*-LOX and Leu439, Leu430, Leu575, and Thr610 of *A. violaceum* 15*S*-LOX.

**a** **
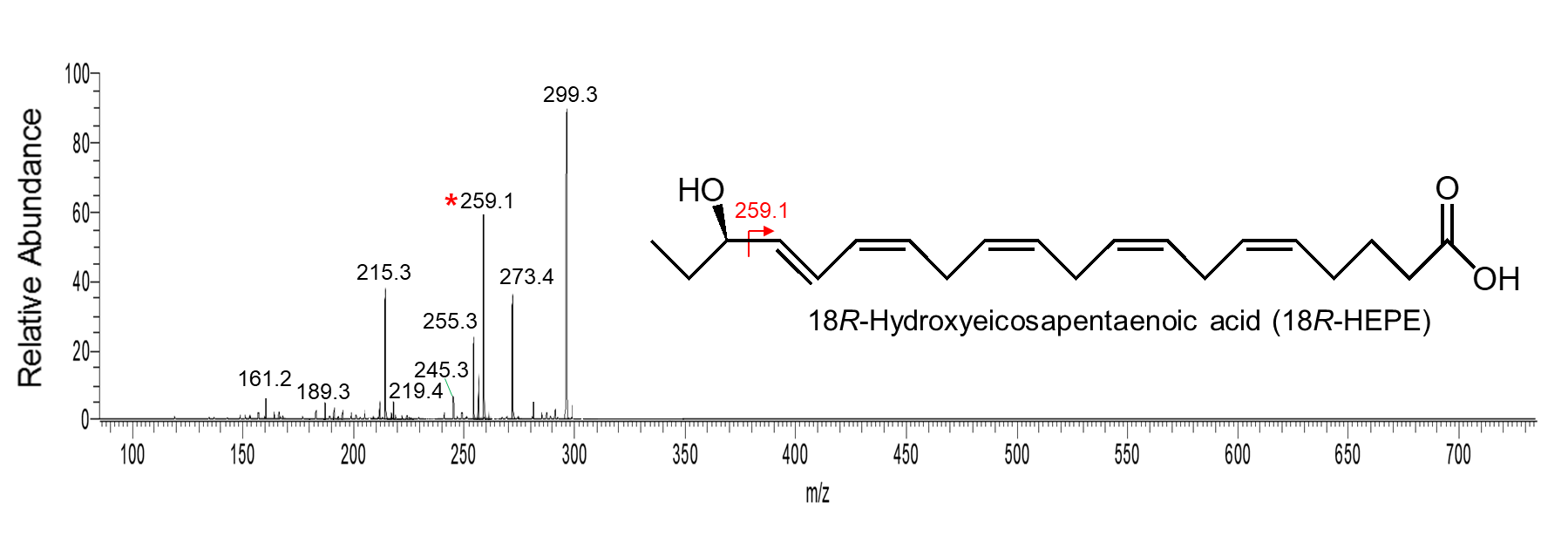
**

**b**

**
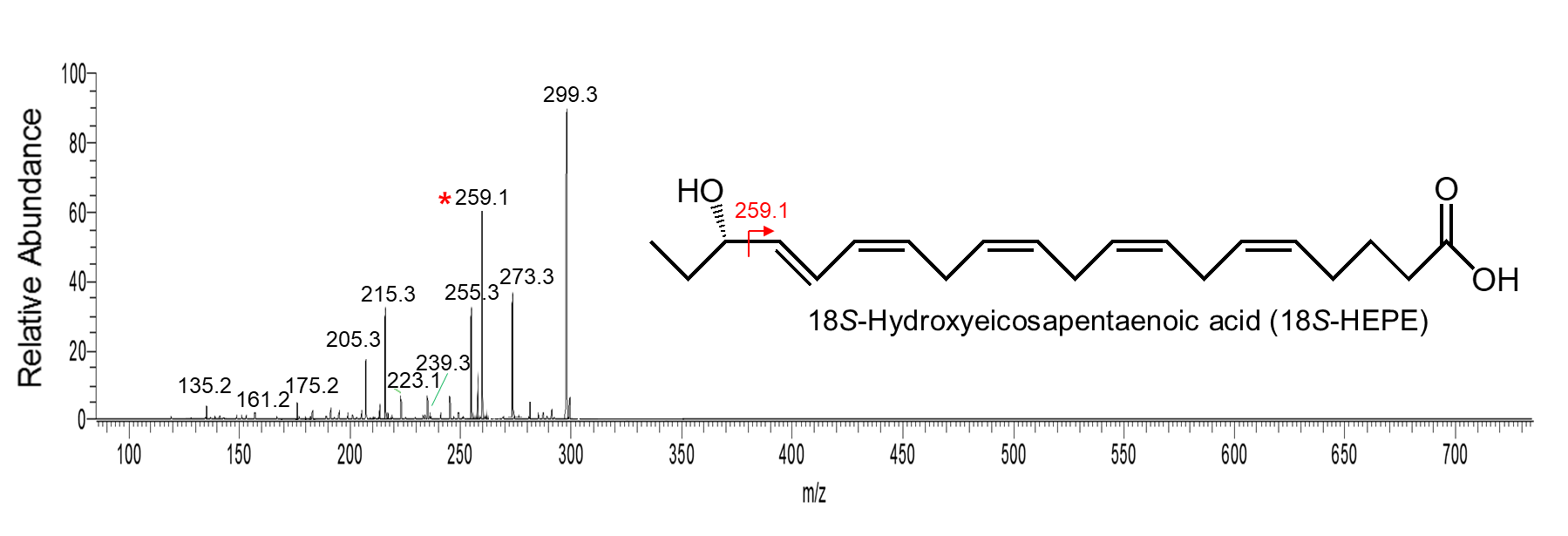
**

**Figure S3.** LC-MS/MS chromatograms of 18*R*- and 18*S*-HEPEs obtained from the conversion of EPA by engineered 18*R*-LOX from *S. cellulosum* and engineered 18*S*-LOX from *A. violaceum*, respectively. (a) LC-MS/MS chromatogram of 18*R*-HEPE. (b) LC-MS/MS chromatogram of 18*S*-HEPE. The red-colored marks indicate critical fragments.

**a** **
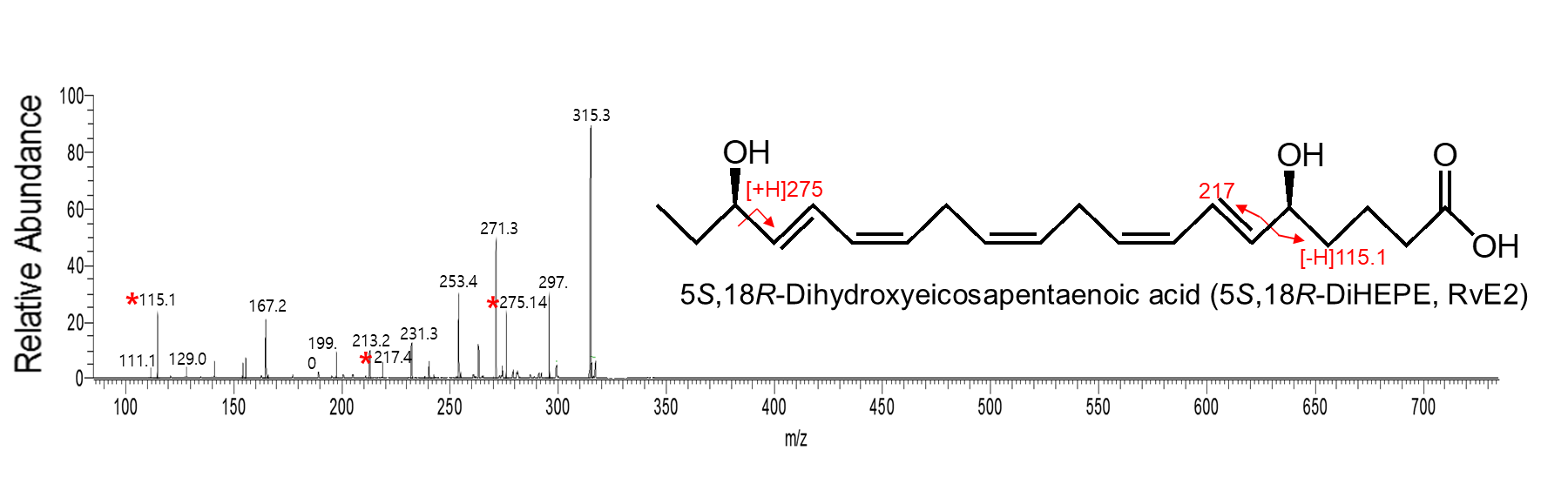
**

**b**

**
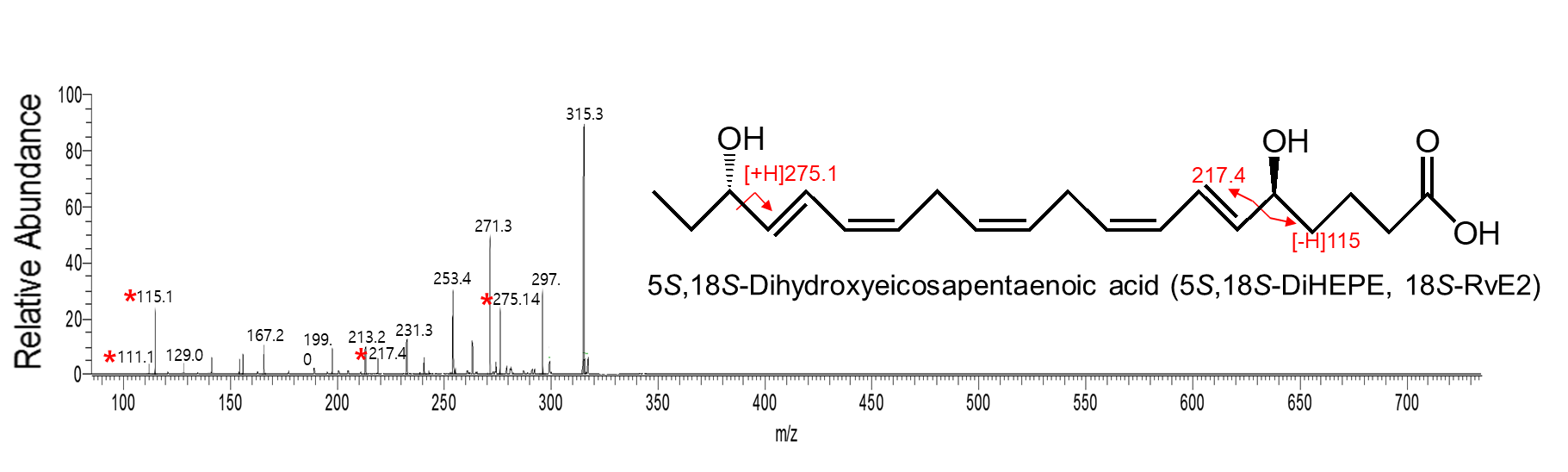
**

**Figure S4.** LC-MS/MS chromatograms of 5*S*,18*R*-DiHEPE (RvE2) and 5*S*,18*S*-DiHEPE (18*S*-RvE2) obtained from the conversion of 18*R*-HEPE and 18*S*-HEPE by 5*S*-LOX from *Danio rerio*, respectively. (a) LC-MS/MS chromatogram of RvE2. (b) LC-MS/MS chromatogram of 18*S*-RvE2. The red-colored marks indicate critical fragments.

**a**

**b**

**Figure S5.** Effects of pH and temperature on 18*R*-HEPE production from EPA by *E. coli* expressing engineered 18*R*-LOX from *S. cellulosum*. (**a**) Effect of pH. The reactions were performed at a constant temperature of 20 ℃ in 50 mM HEPES (*●*, pH 7.0−8.0), 50 mM EPPS (*○*, pH 7.5−8.5) and 50 mM CHES buffers (▼, pH 8.5−9.5) containing 1.0 g/L cells, 1.0 mM EPA, and 200 mM cysteine by ranging the pH from 7.0 to 9.5 for 15 min. (**b**) Effect of temperature. The reactions were performed at a constant pH of 7.5 in 50 mM HEPES buffer retaining 1.0 g/L cells, 1.0 mM EPA, and 200 mM cysteine by varying the temperature from 15 to 35 ℃ for 15 min. Error bars and data indicate the standard deviation and means of three experiments, respectively.

**a**

**b**

**Figure S6.** Optimization of pH and temperature for 18*S*-HEPE production from EPA by *E. coli* expressing engineered 18*S*-LOX from *A. violaceum*. (**a**) Effect of pH. The reactions were performed at a constant temperature of 20 ℃ in 50 mM HEPES (*filled circle*, pH 7.0−8.0), 50 mM EPPS (*empty circle*, pH 7.5−8.5) and 50 mM CHES buffers (*filled-inverted triangle*, pH 8.5−9.5) containing 1.0 g/l cells, 1.0 mM EPA, and 200 mM cysteine by ranging the pH from 7.0 to 9.5 for 15 min. (**b**) Effect of temperature. The reactions were performed at a constant pH of 8.5 in 50 mM EPPS buffer retaining 1.0 g/l cells, 1.0 mM EPA, and 200 mM cysteine by varying the temperature from 15 to 35 ℃ for 15 min. Error bars and data indicate the standard deviation and means of three experiments, respectively.

**a**

**b**

**Figure S7.** Optimization of substrate and cell concentrations for 18*R*-HEPE production by *E coli* expressing engineered 18*R*-LOX from *S. cellulosum*. (**a**) Effect of substrate concentration. (**b**) Effect of cell concentration. The reactions were performed at 20 ℃ in 50 mM HEPES (pH 7.5) containing 1.0 g/l cells and 200 mM cysteine by varying the concentration of EPA from 0.5 to 5.0 mM or the cell concentration from 0.5 to 8.0 g/l for 30 min. Error bars and the data indicate the standard deviation and means of three experiments, respectively.

**a**

**b**

**Figure S8.** Optimization of substrate and cell concentrations on 18*S*-HEPE production by *E coli* expressing engineered 18*S*-LOX from *A. violaceum*. (**a**) Effect of substrate concentration. (**b**) Effect of cell concentration. The reactions were performed at 20 ℃ in 50 mM EPPS (pH 8.5) containing 1.0 g/l cells and 200 mM cysteine by varying the concentration of EPA from 0.5 to 5.0 mM or the cell concentration of 0.5 to 8.0 g/l for 30 min. Error bars and the data indicate the standard deviation and means of three experiments, respectively.

**a**

**b**

**Figure S9.** Optimization of substrate concentration on resolvin E2 (RvE2) and 18*S*-RvE2 production from 18*R*- and 18*S*-HEPEs by *E. coli* expressing *D. rerio* 5*S*-LOX, respectively. (**a**) Effect of substrate concentration on RvE2 production. The substrate 18*R*-HEPE was obtained from the conversion of EPA by *S. cellulosum* engineered 18*R*-LOX. (**b**) Effect of substrate concentration on 18*S*-RvE2 production. The substrate 18*S*-HEPE was obtained from the conversion of EPA by *A. violaceum* engineered 18*S*-LOX. The reactions were performed at 20 ℃ in 50 mM HEPES buffer (pH 8.0) containing 1.0 g/l enzyme, and 200 mM cysteine by ranging the substrate concentration from 0.1 to 1.0 mM for 60 min.

**Supplement Tables**

**Table S1**. Primer design for amplification of specific DNA sequences

| Template | Name | Type | Sequence (5'→3') |
| --- | --- | --- | --- |
| *S. cellulosum* 15*R-*LOX | L423W | Forward | cccggtggacaactggctcgccgggacg |
|  |  | Reverse | cgtcccggcgagccagttgtccaccggg |
|  | L424W | Forward | ccggtggacaacctgtgggccgggacgatcgcg |
|  |  | Reverse | cgcgatcgtcccggcccacaggttgtccaccgg |
|  | L423W/L424M | Forward | ggtggacaactggatggccgggacgatcg |
|  |  | Reverse | cgatcgtcccggccatccagttgtccacc |
|  | L423W/L424M/L568M | Forward | cggcgctcccgatggccgcctacgc |
|  |  | Reverse | gcgtaggcggccatcgggagcgccg |
|  | L423W/L424W | Forward | gaggcccggtggacaactggtgggccgggacgatcgcgtc |
|  |  | Reverse | gacgcgatcgtcccggcccaccagttgtccaccgggcctc |
|  | L423W/L424Y | Forward | ccggtggacaactggtatgccgggacgatcgcg |
|  |  | Reverse | cgcgatcgtcccggcataccagttgtccaccgg |
|  | L423Y/L424Y | Forward | gcccggtggacaactattatgccgggacgatc |
|  |  | Reverse | gatcgtcccggcataatagttgtccaccgggc |
|  | L423W/L424M/L568F | Forward | cggcgctcccgttcgccgcctac |
|  |  | Reverse | gtaggcggcgaacgggagcgccg |
|  | L423W/L424M/L568V | Forward | cggcgctcccggtcgccgcctac |
|  |  | Reverse | gtaggcggcgaccgggagcgccg |
|  | L423W/L424M/L568M/L603F | Forward | tccagctgaacgtgttcatgctcctcggcgg |
|  |  | Reverse | ccgccgaggagcatgaacacgttcagctgga |
|  | L423W/L424M/L568M/L603M | Forward | gccgaggagcatcaacacgttcagctgga |
|  |  | Reverse | tccagctgaacgtgttgatgctcctcggc |
| *A. violaceum* 15*S*-LOX | L429W | Forward | ctcaccggcgagccactggtccaccggg |
|  |  | Reverse | cccggtggaccagtggctcgccggtgag |
|  | L430W | Forward | cttgatctcaccggcccacagctggtccaccgg |
|  |  | Reverse | ccggtggaccagctgtgggccggtgagatcaag |
|  | L429W/L430M | Forward | ccttgatctcaccggccatccactggtccaccgggcca |
|  |  | Reverse | tggcccggtggaccagtggatggccggtgagatcaagg |
|  | L429W/L430M/L575M | Forward | gtaggccgccatcggcagcgccg |
|  |  | Reverse | cggcgctgccgatggcggcctac |

**Table S2**. Regression equations for calibration curves of MonoHFAs and DiHFAs

| **Type** | **Compound** | **Regression equation*^a,b^*** | ***r*^2^** |
| --- | --- | --- | --- |
| MonoHFAs | 15*R*-HEPE | *y* = 0.00009961*x* − 0.0008 | 0.9820 |
|  | 15*S*-HEPE | *y* = 0.000192*x* + 0.0015 | 0.9768 |
|  | 18*R*-HEPE | *y* = 0.0000801*x* − 0.0007 | 0.9780 |
|  | 18*S*-HEPE | *y* = 0.0000882*x* + 0.0015 | 0.9961 |
| DiHFAs | 5*R*,15*R*-DiHEPE | *y* = 0.00006181*x* + 0.0002 | 0.9899 |
|  | 5*S*,15*S*-DiHEPE | *y* = 0.00006081*x* + 0.0009 | 0.9910 |
|  | 5*S*,18*R*-DiHEPE | *y* = 0.00007014*x* + 0.0012 | 0.9807 |
|  | 5*S*,18*S*-DiHEPE | *y* = 0.00007514*x* + 0.0018 | 0.9778 |

MonoHFAs, monohydroxy fatty acids; DiHFAs, dihydroxy fatty acids; HEPE, hydroxyeicosapentaenoic acid ; DiHEPE, dihydroxyeicosapentaenoic acid.

*^a^ x*, peak area in the HPLC profile; *y*, molar concentration of standard (mM). *^b^* Data represent the mean ± SD (n = 3) values.
